# Terahertz time-domain attenuated total reflection spectroscopy integrated with a microfluidic chip

**DOI:** 10.3389/fbioe.2023.1143443

**Published:** 2023-03-13

**Authors:** Ying Fu, Tunan Chen, Ligang Chen, Yuansen Guo, Zhongbo Yang, Ning Mu, Hua Feng, Mingkun Zhang, Huabin Wang

**Affiliations:** ^1^ Center of Super-Resolution Optics & Chongqing Engineering Research Center of High-Resolution and Three-Dimensional Dynamic Imaging Technology, Chongqing Institute of Green and Intelligent Technology, Chinese Academy of Sciences, Chongqing, China; ^2^ Chongqing School, University of Chinese Academy of Sciences, Chongqing, China; ^3^ Department of Neurosurgery and Key Laboratory of Neurotrauma, Southwest Hospital, Third Military Medical University (Army Medical University), Chongqing, China

**Keywords:** terahertz, attenuated total reflection, microfluidic chip, evanescent field, lactate dehydrogenase

## Abstract

The integration of a microfluidic chip into terahertz time-domain attenuated total reflection (THz TD-ATR) spectroscopy is highly demanded for the accurate measurement of aqueous samples. Hitherto, however little work has been reported on this regard. Here, we demonstrate a strategy of fabricating a polydimethylsiloxane microfluidic chip (M-chip) suitable for the measurement of aqueous samples, and investigate the effects of its configuration, particularly the cavity depth of the M-chip on THz spectra. By measuring pure water, we find that the Fresnel formulae of two-interface model should be applied to analyze the THz spectral data when the depth is smaller than 210 μm, but the Fresnel formula of one-interface model can be applied when the depth is no less than 210 μm. We further validate this by measuring physiological solution and protein solution. This work can help promote the application of THz TD-ATR spectroscopy in the study of aqueous biological samples.

## 1 Introduction

Terahertz (THz) wave normally refers to the electromagnetic radiation with a frequency ranging from 0.1 THz to 10.0 THz, which is located between microwave and infrared regions (Wang et al., 2020a; Li et al., 2020; Yang et al., 2020; Meng et al., 2023). In the recent 2 decades, THz biological studies have attracted much attention among scientific communities, mainly thanks to the broad-band, label-free, and bio-sensitive features of THz wave (Zaytsev et al., 2015; Zhou et al., 2016; Sun et al., 2018; Bai et al., 2020; Chen et al., 2020; Zhu et al., 2020; Shen J. et al., 2021; Shen S. et al., 2021; Zhang et al., 2022b; Tang et al., 2022; Zhang et al., 2023). THz time-domain attenuated total reflection (THz TD-ATR) spectroscopy is an advanced THz technique, and superior to many conventional THz techniques due to its remarkable sensitivity by taking the advantage of evanescent-field detection (Arikawa et al., 2008; Wang et al., 2019; Hu et al., 2021; Liao et al., 2022). Moreover, THz TD-ATR spectroscopy enables the minimization of THz absorption by water when measuring aqueous samples. As a result, THz TD-ATR spectroscopy is preferred by the researchers probing aqueous biological samples where high detection sensitivity and minimized water absorption are urgently required (Baxter and Guglietta, 2011; McIntosh et al., 2012; Qin et al., 2017; Peng et al., 2021). Employing this technique, various aqueous biological samples such as protein, DNA, amino acids, and saccharide have been investigated, which greatly promoted the development and application of THz TD-ATR spectroscopy in biological studies (Qin et al., 2013; Yang et al., 2016; Wang et al., 2020b; Tang et al., 2020).

THz TD-ATR spectroscopy includes two core parts, i.e., a THz TD spectroscopy system and an ATR prism incorporated into the THz optical path to allow in- and out-coupling of a THz beam. Normally, the prim is made of high-resistivity silicon, which is an almost perfectly transparent, non-dispersive and high-index material in the THz band. In a typical THz TD-ATR spectroscopy experiment, a sample is deposited onto the prism base, and the THz beam incident at a certain angle experiences total internal reflection at the prism-sample interface, producing an evanescent field attenuating exponentially inside the sample along the normal direction of the sample-prism boundary (Nagai et al., 2006; Peng et al., 2020). By detecting and analyzing the reflected detected THz beam, useful information on the measured sample is extracted. To obtain a reliable result, the sample thickness is required to be no less than the penetration depth of the evanescent field which is dependent on the THz frequency and the sample refractive index (Averett et al., 2008; Gotz et al., 2020; Mendoza-Galvan et al., 2021). To meet this requirement, a common practice in the study of aqueous sample is depositing a large amount of sample solution directly onto the ATR prism base or into an open liquid cell situated on the ATR prism base to guarantee the ATR reflection surface to be completely submerged and the thickness of the solution is high enough to prevent the penetration of THz evanescent field (Gu et al., 2019). Although this operation may work in some cases, it has at least two issues: 1) the inevitable solvent evaporation causes changes in the sample concentration, which compromises the accuracy of results; and 2) it needs relatively a large amount of sample, which is not allowed for rare or precious samples. The integration of a microfluidic chip (M-chip) into THz TD-ATR spectroscopy is a promising approach for tackling the above-mentioned issues, simultaneously (Karabudak, 2013). Although a few groups reported the application of M-chips in transmission mode THz spectroscopy (Xu et al., 2017; Gong et al., 2019), little work has been conducted to fabricate suitable M-chips for THz TD-ATR spectroscopy. Usually, M-chips used for transmission mode THz spectroscopy are not applicable for THz TD-ATR spectroscopy, and the mechanisms underlying the interactions between THz wave and M-chips are different for the 2 THz techniques.

In the present work, we fabricate a polydimethylsiloxane (PDMS) M-chip, integrate it into THz TD-ATR spectroscopy to form a THz TD-ATR microfluidic system, and investigate the configuration, particularly the influence of the depth of M-chip cavity on THz spectra. By measuring pure water, we find that the Fresnel formula of two-interface model should be used to extract aqueous sample properties when the cavity depth is smaller than 210 μm, but a simple Fresnel formula of one-interface model can be applied when the cavity depth is no less than 210 μm. This observation is further confirmed by measuring physiological solution and protein solution. Collectively, we demonstrate that the THz-TD ATR microfluidic system can serve as a useful platform for the study of aqueous biological samples.

## 2 Results and discussion

### 2.1 Configuration of microfluidic chips

**FIGURE 1:**
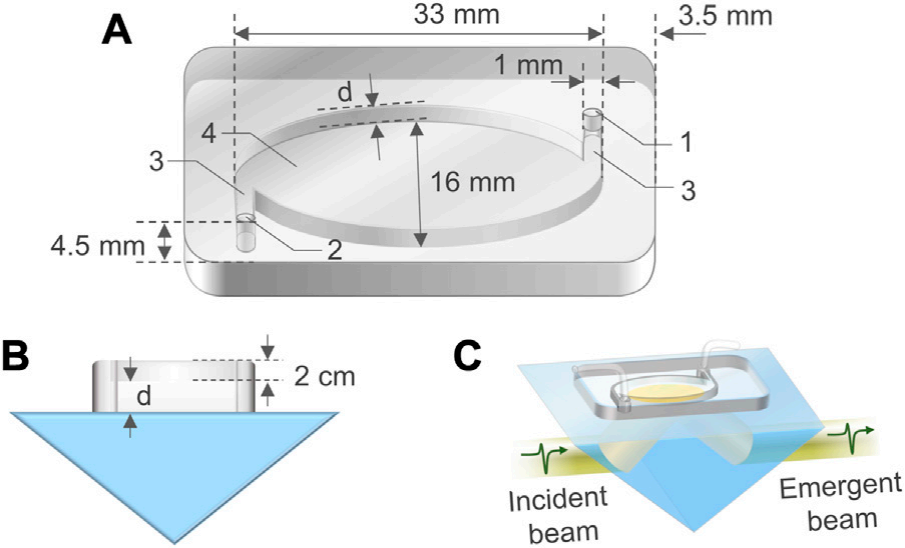
Configuration of a M-chip. **(A)** A 3D schematic view of a M-chip, in which the inlet “1” and outlet “2” holes, channels “3”, and cavity “4” are connected. The diameter of the holes is 1 mm. The cavity is in a shape of elliptic cylinder, with a major axis length of 33 mm, and a minor axis length of 16 mm. The distances between the holes and the chip outside edges are 3.5 mm along the major axis direction and 4.5 mm along the minor axis direction of the cavity, respectively. **(B)** Side view of a M-chip situated on an ATR prism. The depth of channels is the same as the cavity depth ( d ), which is a variable. The depth of the holes is the summation of the cavity depth and the thickness (2 cm) of the upper wall. **(C)** A 3D schematic view of the ATR apparatus integrated with a M-chip. A *p*-polarized THz beam is incident horizontally to the left slanted side of the ATR prism, an isosceles triangulated column, then refracted and incident to the prism base at which the attenuated total internal reflection occurs. The emergent beam is detected for analysis.

PDMS is a popular material for fabricating microfluidic devices due to its advantages such as highly biocompatible, chemical inert, impermeable to water, inexpensive, flexible, and easy to be fabricated and bonded to other surfaces. PDMS M-chips were fabricated *via* injection molding (Sia and Whitesides, 2003) (Notes S1 and S2 of the Supplementary Material). Figure 1A is a three-dimensional (3D) schematic view of a fabricated M-chip, including an inlet, an outlet, a cavity with an elliptic cylinder shape, and two channels tangential to the wall of the cavity. The M-chip has upper wall and side wall, but no bottom (Figure 1B). This design ensures an aqueous sample in the cavity to contact with the ATR prism base without any barriers, enabling the sample to be directly probed with the evanescent filed generated by the total internal reflection occurring at the sample-prism interface (Figure 1C). Before a THz TD-ATR measurement, the M-chip was aligned carefully to make the cavity to fully cover the elliptical THz beam spot, and securely attached and sealed on the prism base by applying an even loading. A syringe or pump was employed to infuse a sample solution through a soft tube into the inlet by a pressure-driven mechanism, and the solution passed through the channel connected with the inlet to fill the cavity. The excess sample solution and air bubbles were drained out through the outlet.

### 2.2 Analytical models for THz TD-ATR spectroscopy integrated with a M-chip

**FIGURE 2:**
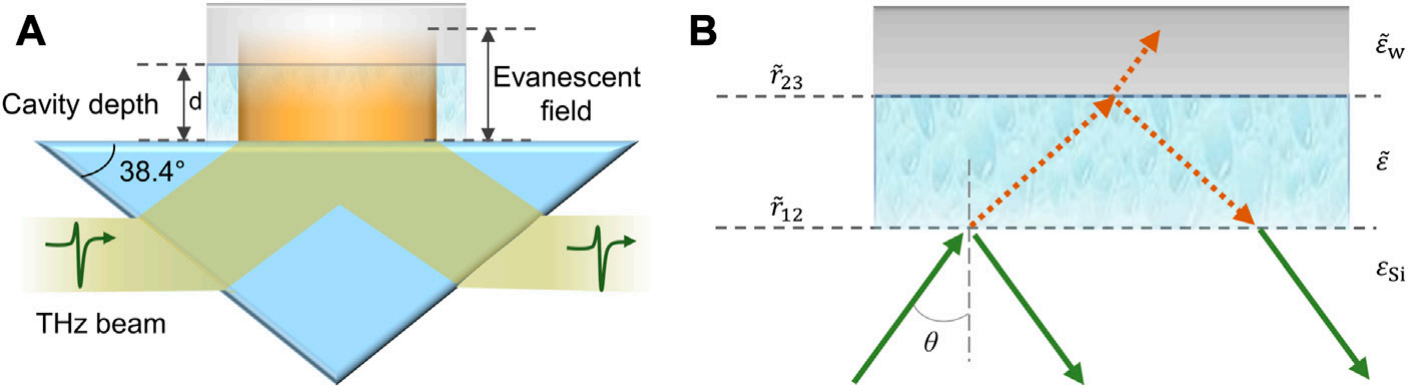
Schematic illustration of the interaction mechanism between the THz evanescent field and sample and M-chip. **(A)** THz evanescent field (in orange color) occurs at the prism-substance interface, penetrates the cavity, and enters the upper wall of the M-chip. The angle of ATR prism slant side is 38.4°. The substance in the cavity of the M-chip can be either air (i.e., reference) or aqueous sample. A prism-substance interface and a substance-PDMS upper-wall interface are formed. As the side wall of the M-chip does not influence the analysis in our case, it is not shown for simplicity. **(B)** ${\tilde{r}}_{12}$ and ${\tilde{r}}_{23}$ are the Fresnel’s reflection coefficients of prism-substance interface and the substance-PDMS upper-wall interface, respectively. ${\tilde{\varepsilon }}_{w}$ , $\tilde{\varepsilon }$ , $\varepsilon_{Si}$ denote the complex dielectric constants of the PDMS, substance in the cavity, and silicon prism, respectively. ${\tilde{E}}_{in}$ and ${\tilde{E}}_{out}$ are the incident and emergent beams, respectively, and $\tilde{r}$ is the ultimate Fresnel’s reflection coefficient of the two-interface system. θ is the incident angle of the THz beam in the prism.

For proper application of the THz TD-ATR spectroscopy integrated with a PDMS M-chip, namely, the THz TD-ATR microfluidics system, we have to carefully consider the possible influence of the configuration of the M-chip on THz spectra. It is a non-trivial work to extract the true THz spectra of an aqueous sample filled in the cavity if the side wall of the M-chip interacts with the evanescent field. Fortunately, for a certain THz TD system, the cross section of the evanescent filed at the sample-prism interface can be estimated. In our case, the diameter of the THz beam is about 12 mm, yielding an elliptical beam spot on the prism base with major axis length of ∼31 mm (12/sin^2^ 38.4°) and minor axis length of ∼12 mm. Thus, we designed a M-chip with a cavity cross section larger than that of the evanescent field. With proper alignment of the M-chip, we ensured only the cavity region overlaid on the evanescent field, by which the influence of the side wall was avoided (Figure 2A). Although the effects of the side wall can be eliminated with this design, the influence of the upper wall of the M-chip has to be taken into account, i.e., the THz reflection occurred at the interface formed by the sample and PDMS upper wall needs to be considered, in order to extract reliable THz spectral data of the sample measured by the THz TD-ATR microfluidics system.

The sample complex dielectric constant is key for analyzing the sample properties. For our THz TD-ATR microfluidic system, the Fresnel’s formulae for two-interface model are used to extract the complex dielectric constant of the sample for general cases (Møller et al., 2007),

$${\tilde{r}}_{12}=\frac{\sqrt{\varepsilon_{Si}}\sqrt{1-\left(\varepsilon_{Si}/\tilde{\varepsilon }\right)\sin^{2}\theta }-\sqrt{\tilde{\varepsilon }}\cos\theta }{\sqrt{\varepsilon_{Si}}\sqrt{1-\left(\varepsilon_{Si}/\tilde{\varepsilon }\right)\sin^{2}\theta }+\sqrt{\tilde{\varepsilon }}\cos\theta }$$ (1)

$${\tilde{r}}_{23}=\frac{\sqrt{\tilde{\varepsilon }}\sqrt{1-\left(\varepsilon_{Si}/{\tilde{\varepsilon }}_{w}\right)\sin^{2}\theta }-\sqrt{{\tilde{\varepsilon }}_{w}}\sqrt{1-\left(\varepsilon_{Si}/\tilde{\varepsilon }\right)\sin^{2}\theta }}{\sqrt{\tilde{\varepsilon }}\sqrt{1-\left(\varepsilon_{Si}/{\tilde{\varepsilon }}_{w}\right)\sin^{2}\theta }+\sqrt{{\tilde{\varepsilon }}_{w}}\sqrt{1-\left(\varepsilon_{Si}/\tilde{\varepsilon }\right)\sin^{2}\theta }}$$ (2)

where ${\tilde{r}}_{12}$ and ${\tilde{r}}_{23}$ are the Fresnel’s reflection coefficients of prism-substance interface and the substance-PDMS upper-wall interface, respectively; ${\tilde{\varepsilon }}_{w}$ , $\tilde{\varepsilon }$ , and $\varepsilon_{Si}$ are the complex dielectric constants of the PDMS, substance filled in the cavity and silicon prism, respectively; and θ is the incident angle of the THz beam in the prism.

For a cavity with a depth of d and an incident wave with a wavelength of λ , the ultimate Fresnel’s reflection coefficient ( $\tilde{r}$ ) that contains the information of the substance in the cavity and PDMS upper-wall due to their interactions with the evanescent wave is calculated by (McIntyre and Aspnes, 1971)

$$\tilde{r}=\frac{{\tilde{r}}_{12}+{\tilde{r}}_{23}\exp\left(i\frac{4\pi d}{\lambda }\sqrt{\tilde{\varepsilon }-\varepsilon_{Si}\sin^{2}\theta }\right)}{1+{\tilde{r}}_{12}{\tilde{r}}_{23}\exp\left(i\frac{4\pi d}{\lambda }\sqrt{\tilde{\varepsilon }-\varepsilon_{Si}\sin^{2}\theta }\right)}$$ (3)

It needs to point out that if the evanescent field cannot penetrate the cavity to reach the upper wall of the M-chip, ${\tilde{r}}_{23}$ is zero, thus ${\tilde{r}}_{12}$ is equal to $\tilde{r}$ . In this case, the two-interface model is degenerated to the one-interface model.

In the experiments, the M-chip without and with an aqueous sample was measured in tandem to obtain the information of the reference (i.e., air) and the sample, respectively. The above three equations are applicable to both the reference and the sample. By substituting Eqs 1, 2 into Eq. 3, the ultimate Fresnel’s reflection coefficients of the reference ( ${\tilde{r}}_{ref}$ ) and the sample ( ${\tilde{r}}_{sam}$ ) were obtained, respectively. Furthermore, the relationship between the ultimate reflected electromagnetic field amplitudes of the sample ( ${\tilde{E}}_{sam}$ ) and reference ( ${\tilde{E}}_{ref}$ ) from the prism-substance interface and their corresponding reflection coefficients can be described by Eq. 4,

$$\frac{{\tilde{E}}_{sam}}{{\tilde{E}}_{ref}}=\frac{{\tilde{r}}_{sam}}{{\tilde{r}}_{ref}}$$ (4)

In the above equations, $\varepsilon_{Si}$ (3.42), d ; λ , θ (51.6°) and the dielectric constant of air (1) are all known, and ${\tilde{\varepsilon }}_{w}$ was measured using the THz TD spectroscopy in transmission mode (Supplementary Figure S3). With the above information, the complex dielectric constant of the measured sample can be calculated.

THz absorption coefficient ( α ) is also often used to describe the properties of samples, which is calculated according to the following equations (Jepsen et al., 2011; Zhang et al., 2022a),

$$\tilde{\varepsilon }\left(\nu \right)=\varepsilon^{\prime }\left(\nu \right)-i\varepsilon^{\prime \prime }\left(\nu \right)$$ (5)

$$\varepsilon^{\prime }\left(\nu \right)=n^{2}\left(\nu \right)-k^{2}\left(\nu \right)$$ (6)

$$\varepsilon^{\prime \prime }\left(\nu \right)=2n\left(\nu \right)k\left(\nu \right)$$ (7)

$$\alpha \left(\nu \right)=\frac{4\pi \nu k\left(\nu \right)}{c}$$ (8)

where $\varepsilon^{\prime }$ and $\varepsilon^{\prime \prime }$ are the real and imaginary part of $\tilde{\varepsilon }$ , respectively; ν is the frequency; n and k are the refractive index and extinction coefficient, respectively; and c is the speed of light in vacuum.

### 2.3 Dielectric measurement of pure water using the THz TD-ATR microfluidic system

**FIGURE 3:**
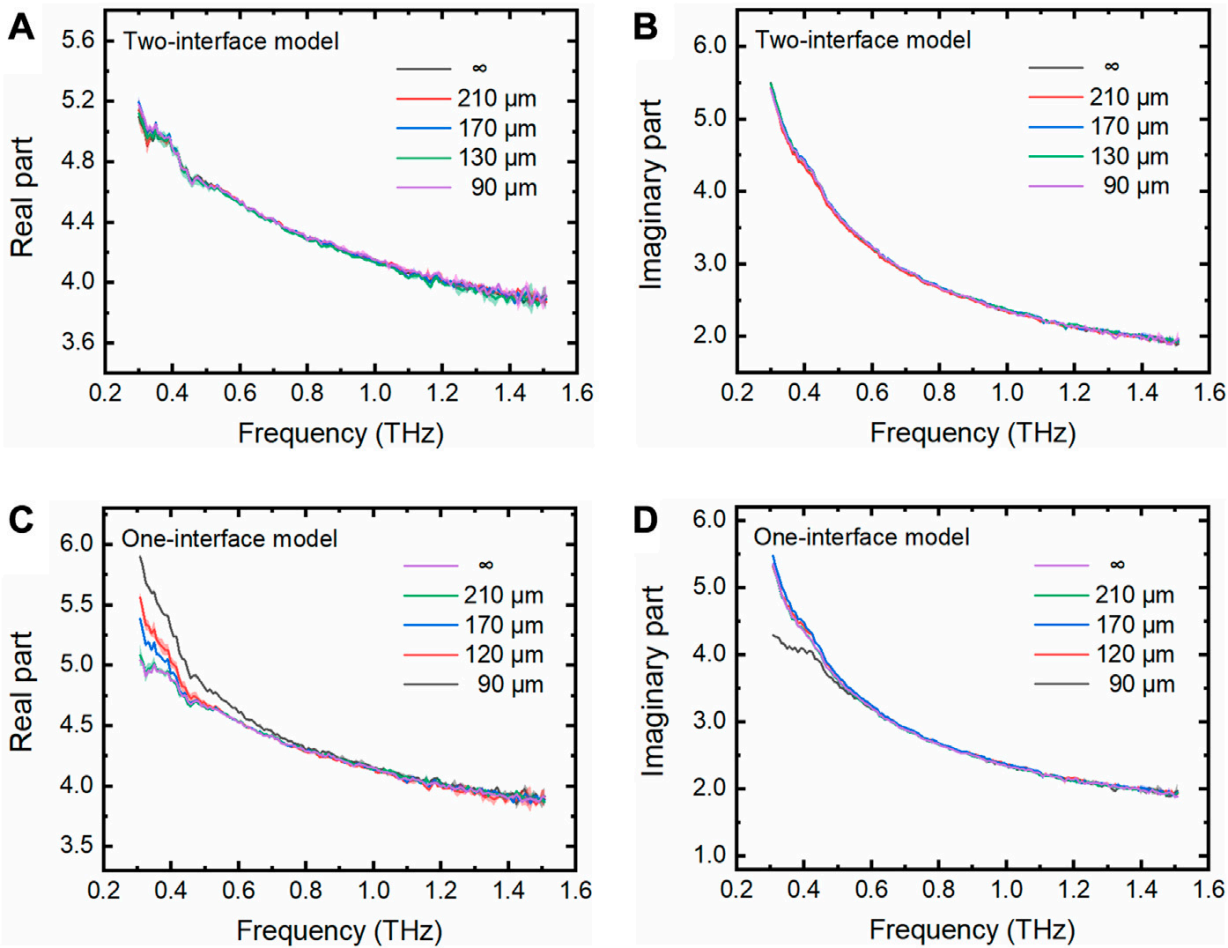
THz spectra of pure water measured using the THz TD-ATR microfluidic system. **(A–B)** Real and imaginary parts of the complex dielectric constants of pure water filled in the M-chips with various depths (90, 130, 170, and 210 μm) and the infinite thickness relative to the penetration depth of the evanescent waves, denoted by “∞”, which were extracted by the two-interface model. **(C–D)** These parameters were extracted by the one-interface model. The semitransparent homochromic shadows around the lines represent the standard deviation (SD) of three independent measurements.

Pure water is commonly used as the solvent for many biological samples. Therefore, we measured the THz spectra of pure water (Milli-Q water, 18.2 MΩ cm) using our fabricated M-chips with various cavity depths. By applying the two-interface model, we can see that the complex dielectric constants of pure water measured in the M-chips of various depths are highly consistent with each other (Figures 3A,B). They are also highly consistent with those measured without using the M-chips, for pure bulk water through which the THz evanescent field cannot penetrate. The results tell us that the two-interface model is accurately enough to be used to extract the THz properties of pure water in the cavity of M-Chips.

In contrast, the complex dielectric constants are not necessarily consistent with each other when the one-interface model was applied to analyze the data. Although the complex dielectric constant of pure water measured in the cavity with a depth of 210 μm is consistent with that of pure bulk water, the parameters extracted for the pure water in the cavities with depths of 90, 130, and 170 μm deviate from those of pure bulk water at the lower THz frequency range (Figures 3C,D). These observations indicate that the evanescent field penetrated the pure water with a thickness less than 210 μm, and reached the upper wall of the PDMS M-chip. Obviously, it is inappropriate to apply the one-interface model when the cavity depth is less than 210 μm because the one interface model is only suitable for the situation where the evanescent field does not penetrate the pure water. The influence of the cavity depth on the detected THz wave was further explored by analyzing the depth-related term contained in Eq. 3 for the two-interface model, as shown below,

$$\tilde{\Phi }={\tilde{r}}_{23}\exp\left(i\frac{4\pi d}{\lambda }\sqrt{\tilde{\varepsilon }-\varepsilon_{Si}\sin^{2}\theta }\right)$$ (9)

This term is associated with the cavity depth when the evanescent wave reaches the upper wall of the PDMS M-chip. However, this term is zero, so the two-interface is degenerated to the one-interface model when the evanescent wave cannot penetrate through the aqueous sample in the cavity to reach the upper wall of the PDMS M-chip.

**FIGURE 4:**
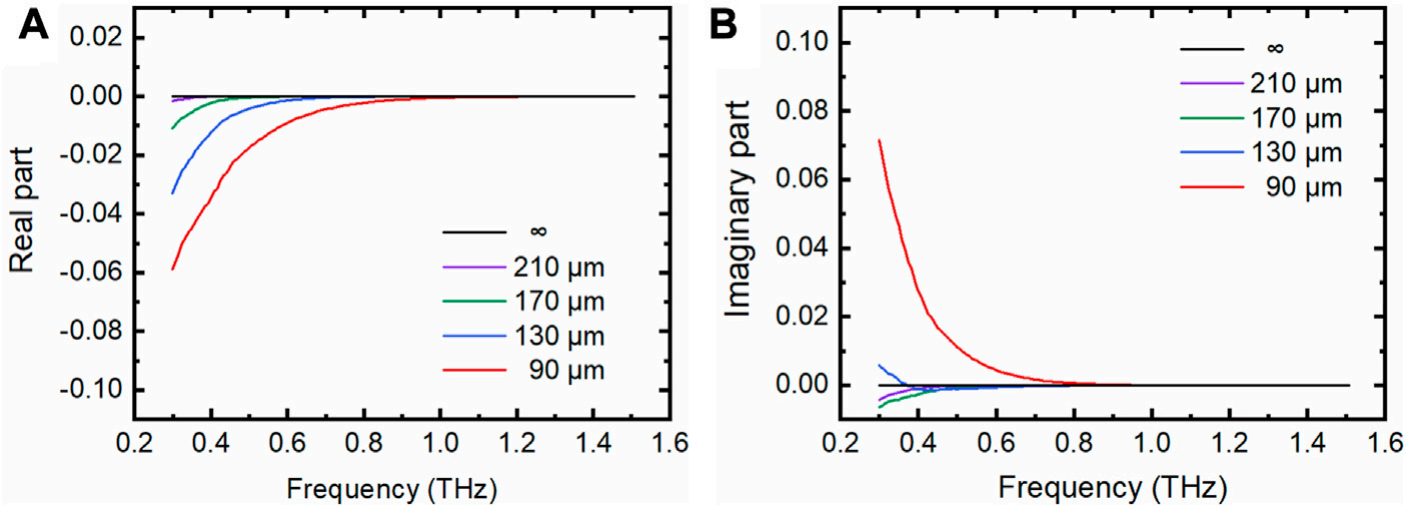
The real parts and imaginary parts of the depth-related term. **(A)** Real parts of the depth-related term $\tilde{\Phi }$ . **(B)** Imaginary parts of $\tilde{\Phi }$ . The cavity depth (pure water thickness) includes 90, 130, 170, and 210 μm “∞” indicates the thickness of pure bulk water, for which either the real part or the imaginary part of $\tilde{\Phi }$ is zero for the frequency range investigated.

Experimentally, indeed, we observed that both the real and imaginary parts of $\tilde{\Phi }$ show a cavity-depth and frequency dependent character, particularly for the lower frequency range (Figures 4A,B). Interestingly, it is found that both the real and imaginary parts of $\tilde{\Phi }$ for the pure water with a thickness of 210 μm are nearly the same as those of the pure bulk water for the frequency is higher than 0.3 THz. The result verifies that the two-interface model can be degenerated to the one-interface model when the pure water thickness is no less than 210 μm, and that the two-interface model should be applied for pure water with the thickness less than 210 μm. These observations well explain the phenomena observed for the dielectric complex constants in Figure 3. For a commonly used THz TD-ATR spectroscopy system, the THz signal below 0.3 THz is very noisy and often not included in the data analysis. Thus, we recommend to use a M-chip with a cavity depth no less than 210 μm in THz TD-ATR microfluidic system, by which one can employ the simple one-interface model to analyze the data with enough reliability while avoid the complexity of the two-interface model. Specifically, the one-interface model is also applicable for M-chips with cavity depths of 90 μm and above when only the frequency beyond ∼1.0 THz is considered in data analysis (Figure 4).

### 2.4 Dielectric measurement of physiological solution using the THz TD‐ATR microfluidic system

**FIGURE 5:**
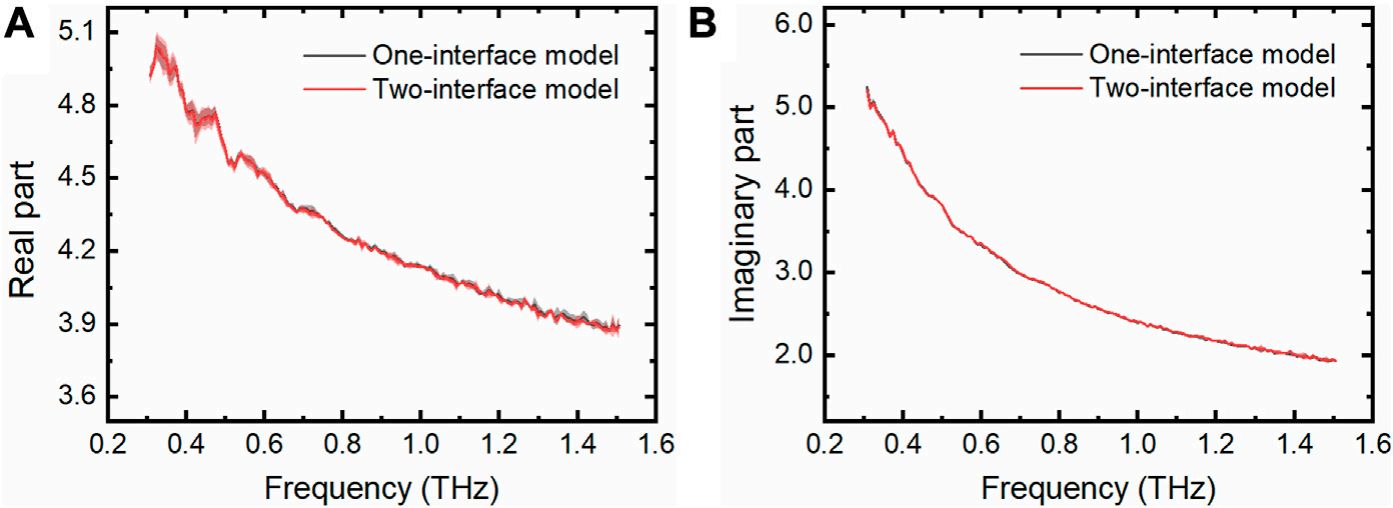
THz spectra of PBS measured by the THz TD-ATR microfluidic system. Real parts **(A)** and imaginary parts **(B)** of the complex dielectric constants of PBS were extracted by employing the one-interface model and the two-interface model, respectively. Translucent areas around each line indicate the standard deviation from three independent tests.

From the above results we know that the THz TD-ATR spectroscopy integrated with a M-chip with a cavity depth no less than 210 μm can be reliably applied to measure pure water by simply using the one-interface model in the data analysis. To test the wide applicability of the THz TD-ATR system of the above configuration, we measured the THz spectra of phosphate buffered saline (PBS) solution, which is a physiological solution widely used to dissolve biological samples. It is evident that the extracted complex dielectric constants obtained by the one-interface model are well superimposed on those retrieved by the two-interface model (Figure 5).

### 2.5 THz absorption measurement of protein solutions using the THz TD-ATR microfluidic system

**FIGURE 6:**
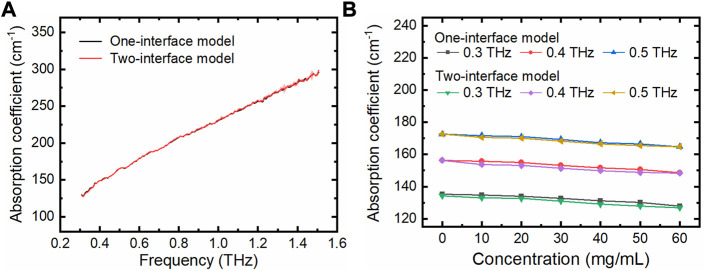
LDH solutions measured using the THz TD-ATR microfluidic system. **(A)** THz absorption coefficients of LDH solution (60 mg/mL) are plotted against the THz frequency, which were extracted using the two-interface model and one-interface model, respectively. **(B)** THz absorption coefficients of LDH solutions at specific THz frequencies are plotted against the LDH concentrations. The solution with a LDH concentration of 0 mg/mL is PBS solution. The error bars for the three independent experiments are too small to be clearly observed.

One of our major purposes is to integrate a suitable M-chip into THz TD-ATR spectroscopy to study biological samples. Thus, we further measured lactic dehydrogenase (LDH) solutions with different concentrations using the PDMS M-chip with a cavity depth of 210 μm, and extracted their THz absorption coefficients which are widely used to assess the character of biological solutions. The THz coefficients were obtained by both the two-interface model and the one-interface model. For example, the THz coefficient of LDH solution with a concentration of 60 mg/mL plotted against a wide-band frequency was shown in Figure 6A. It is clear that for this M-chip configuration, the one-interface model is as accurate as the two-interface model for the measurement of LDH solutions. In addition, we also checked the THz absorption coefficients against the LDH concentrations at certain frequencies (e.g., 0.3, 0.4, and 0.5 THz; Figure 6B). Again, it confirms that there are no observable differences when applying the two models to analyze protein solutions measured by our THz TD-ATR microfluidic system. The THz absorption coefficient decreases with the increase of LDH concentration observed here is consistent with previous studies (Bye et al., 2014), which is very likely due to the solute (LDH) induced dilution and static depolarization effects (Penkov et al., 2017).

## 3 Conclusion

In this work, we demonstrated a simple and low-cost method of fabricating a M-chip for the measurement of a liquid phase sample with the THz TD-ATR. With the integration of the M-chip into the THz TD-ATR, the sample volume could be reduced to an unprecedentedly small volume as less as 40 μL. We also rationalized the application of theoretical models for analyzing the data obtained by the THz TD-ATR microfluidic system, which is critical for the proper application of the system. By measuring pure water, we found that the one-interface model can be safely applied in the data analysis when the cavity depth of M-chips is no less than 210 μm, but the two-interface model should be applied when the depth is less than 210 μm. Based on these results, we propose to use PDMS M-chips with a cavity depth of at least 210 μm in a THz TD-ATR microfluidic system when measuring readily available or cheap aqueous sample solutions to avoid the complexity of using the two-interface model in data analysis. Instead, PDMS M-chips with a cavity depth smaller than 210 μm and the two-interface model are suggested to be applied when measuring precious or expensive aqueous sample solutions in order to save the samples while keep the experimental reliability. With such a system, we successfully detected physiological (PBS) and protein (LDH) solutions. We also note that the theories shown here also hold for other solutions as far as the solutions are inert to PDMS, and that the threshold value of the cavity depth for applying the one-interface model may change, dependent on the performance of the THz TD-ATR and the refractive index of the measured solution (Liu et al., 2017). Collectively, the work presented here is helpful for researchers who exploit THz TD-ATR spectroscopy to investigate aqueous biological samples in a reliable and economical way.

## 4 Methods

### 4.1 Chemicals

Lactic dehydrogenase powder was purchased from Beijing Solarbio Science & Technology Co., Ltd. (Beijing, China), and dissolved in phosphate buffer saline (PBS, pH 7.4, Shanghai Branch of Thermo Fisher Scientific Inc. Shanghai, China) and diluted to desired concentrations with PBS. The prepolymer and the crosslinker of PDMS were ordered from the Shanghai Branch of Dow Corning Corp. (Shanghai, China).

### 4.2 Experimental setup

The THz-TD ATR spectroscopy system was established by incorporating an ATR apparatus (BATOP GmbH, Jena, Germany) into the optical path of a commercial THz-TDS system (Tera K15, Menlo Systems GmbH, Münich, Germany). A femtosecond laser with a center wavelength of 1,560 nm, a repetition rate of 100 MHz, and a pulse width less than 90 fs was split into the pump beam and the probe beam. The THz radiation was emitted from a biased photoconductive antenna irradiated by the pump beam, and detected by another photoconductive antenna with the aid of the probe beam. Before experiments, the microfluidic chip fabricated by us was integrated into the system for the measurement of aqueous samples. The whole system was located in a clean room maintained at a temperature of 22°C ± 1.0°C. In experiments, the apparatus including the THz emitter, ATR prism integrated with microfluidic chip and THz detector were enclosed in a sealed container filled with pure nitrogen and kept the humidity to less than 4% to minimize the vapor absorption of THz wave. A *p*-polarized pulsed THz beam was horizontally incident on the left slanted side of the prism, and the refracted beam was detected from the right side of the prism. The ATR prism and PDMS chip can be re-used after cleaning by ethanol and distilled water in order. Either a milliliter medical syringe (Minkang Medical Materials Co., Ltd. Changsha, China) or a pressure-based flow controller (MFCS-EZ, Fluigent, Paris, France) was used to infuse sample solution into an M-chip.

### 4.3 Data analysis

Time-domain spectra were measured and converted to frequency-domain data by the fast Fourier transform algorithm, from which the electric field amplitude at different frequencies can be obtained. Then the complex dielectric constant and absorption coefficient can be calculated according to Eqs 1–8. The data for each sample represents the average value obtained from three independent measurements.

### 4.4 Fabrication of the microfluidic chip

Microfluidic chips with different cavity depths were fabricated by using a standard protocol, and the details were described in the Supplementary Material.

## Data Availability

The original contributions presented in the study are included in the article/Supplementary Material, further inquiries can be directed to the corresponding authors.
