# Supplementary material for: Terahertz time-domain attenuated total reflection spectroscopy integrated with a microfluidic chip
Source: Front Bioeng Biotechnol. 2023 Mar 13;11:1143443. doi: 10.3389/fbioe.2023.1143443 (PMC10040880; doi:10.3389/fbioe.2023.1143443)
Supplement: Supplementary file 1 [file DataSheet1.pdf]

Supplementary Material

Terahertz time-domain attenuated total reflection spectroscopy integrated with a microfluidic chip

Ying Fu, Tunan Chen, Ligang Chen, Yuansen Guo, Zhongbo Yang, Ning Mu, Hua Feng, Mingkun Zhang*, Huabin Wang*

*** Correspondence:** zhangmk@cigit.ac.cn (M.Z.), wanghuabin@cigit.ac.cn (H.W.)

1 Supplementary Notes

1.1 Note S1. Fabrication of microfluidic chips

Polydimethylsiloxane (PDMS) microfluidic chips with a series of depths of 90, 130, 170 and 210 μm were fabricated via photolithography and injection molding by the following steps. First, the pattern of the mask was designed, according to the configuration of the microfluidic chip needed. In the design, it was ensured that the cavity can fully cover the spot of the elliptical evanescent wave on the reflection surface of the ATR prism. Second, the mask of the microfluidic chip was fabricated via the laser direct writing lithography (DWL66FS, Heidelberg Engineering GmbH., Germany) on a chrome glass plate, in which the chrome layer was removed to form a pattern as the same as the bottom of the cavity of the microfluidic chip. Third, SU-8 2000 negative photoresist was spin-coated on a quartz plate and baked to form a layer with the same thickness as the cavity depth of the desired microfluidic chip. Fourth, the photoresist layer was covered by the mask and treated by the lithography before the photoresist developer was applied on it to make the quartz-photoresist template of the microfluidic chip. Fifth, the prepolymer and the crosslinker of PDMS were uniformly mixed and evenly perfused on the template to form a sheet with a thickness of ~2 cm, which was then allowed to solidify in the oven at 60 ℃ for 6 h. Finally, the PDMS microfluidic chip was achieved by removing the template and punching inlet and outlet holes at designed locations on the PDMS sheet (Figure S2), and integrated into the THz TD-ATR spectroscopy.

1.2 Note S2. Measurement of the dielectric constant of bulk PDMS slaps

Bulk PDMS slabs of 1.16 mm thick were measured by the transmission mode THz TD spectroscopy, and the real (ε') and imaginary (ε'') parts of the dielectric constant were calculated according to following equations:

$$\frac{\tilde{E}_{\mathrm{s}}(\nu)}{\tilde{E}_{\mathrm{r}(\nu)}} = A(\nu)e^{i\varphi(\nu)} \quad (S1)$$

$$n(\nu) = \frac{\varphi(\nu)c}{2\pi\nu d} + 1 \quad (S2)$$

$$\kappa(\nu) = \frac{c}{2\pi\nu d}\ln\frac{4n(\nu)}{A(\nu)[n(\nu)+1]^2} \tag{S3}$$

$$\varepsilon'(\nu) = n^2(\nu) - \kappa^2(\nu) \tag{S4}$$

$$\varepsilon''(\nu) = 2n(\nu)\kappa(\nu) \tag{S5}$$

where $\tilde{E}_s$ and $\tilde{E}_r$ are the electric field of sample and reference, respectively; A and φ are the magnitude ratio and phase shift of the transmitted sample signal to reference signal, respectively; n and k are the refractive index and extinction coefficient, respectively; ν, c and d are the spectral angular frequency, the speed of light in vacuum, and the thickness of PDMS, respectively.

2 Supplementary Figures

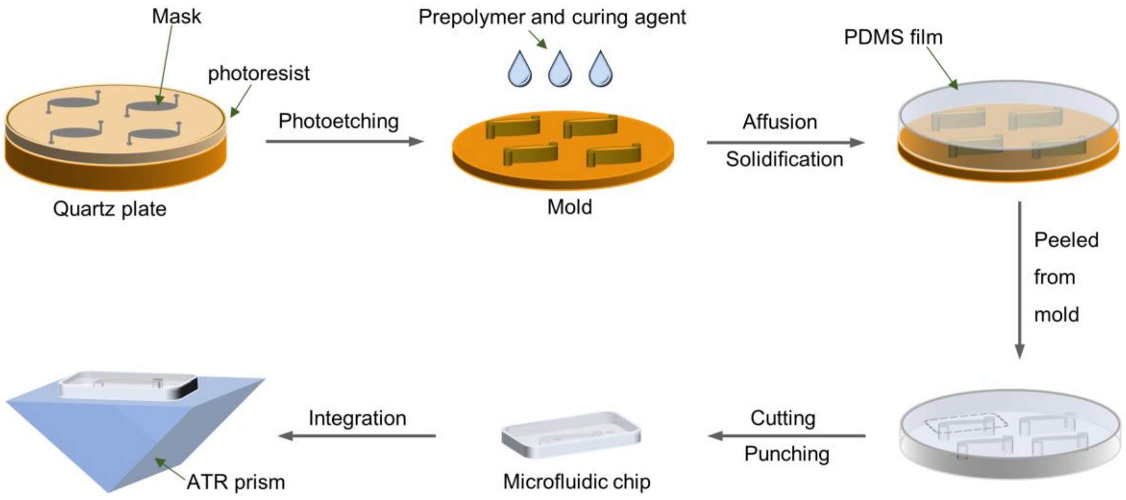

Figure S1. Schematic diagram of the processing procedures of PDMS microfluidic chips.

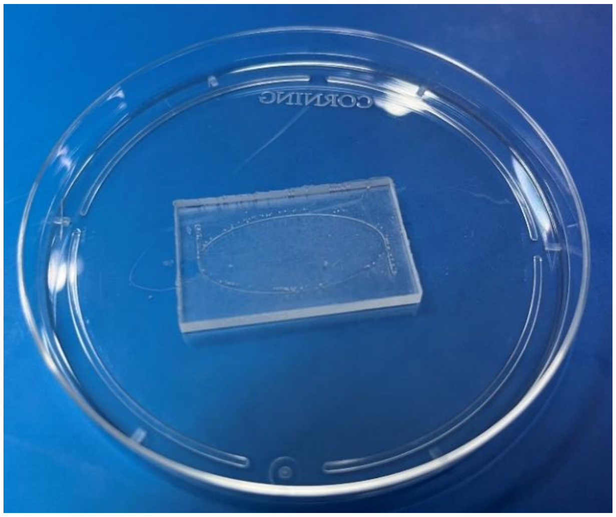

Figure S2. A microfluidic chip sample placed in a petri-dish

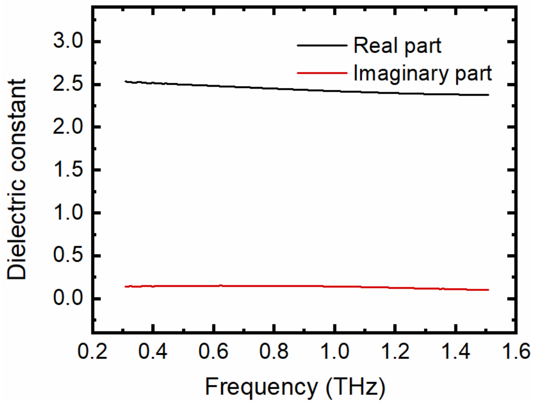

Figure S3. Complex dielectric constant of a bulk PDMS sheet as function of THz frequency. The thickness, length, and width of the slab are 1.16, 40, and 25 mm, respectively. Each line represents the mean of three independent tests, and the standard deviations are too small to be clearly observed.
